# The Hypoxic Landscape Stratifies Gastric Cancer Into 3 Subtypes With Distinct M6a Methylation and Tumor Microenvironment Infiltration Characteristics

**DOI:** 10.3389/fimmu.2022.860041

**Published:** 2022-06-21

**Authors:** Zhi-kun Ning, Ce-gui Hu, Jiang Liu, Hua-kai Tian, Zhong-lin Yu, Hao-nan Zhou, Hui Li, Zhen Zong

**Affiliations:** ^1^ Department of Day Ward, The First Affiliated Hospital of Nanchang University, Nanchang, China; ^2^ Department of Gastrointestinal Surgery, The Second Affiliated Hospital of Nanchang University, Nanchang, China; ^3^ Queen Marry College, Nanchang University, Nanchang, China; ^4^ Department of Rheumatology and Immunology, The First Affiliated Hospital of Nanchang University, Nanchang, China

**Keywords:** hypoxia, m6A, gastric cancer, immune infiltration, immune checkpoint blockade

## Abstract

The interaction between hypoxia and RNA N6-methyladenosine (m6A) is an emerging focus of investigation. However, alterations in m6A modifications at distinct hypoxia levels remain uncharacterized in gastric cancer (GC). Unsupervised hierarchical clustering was performed to stratify samples into different clusters. Differentially expressed gene analysis, univariate Cox proportional hazards regression analysis, and hazard ratio calculations were used to establish an m6A score to quantify m6A regulator modification patterns. After using an algorithm integrating Least absolute shrinkage and selection operator (LASSO) and bootstrapping, we identified the best candidate predictive genes. Thence, we established an m6A-related hypoxia pathway gene prognostic signature and built a nomogram to evaluate its predictive ability. The area under the curve (AUC) value of the nomogram was 0.811, which was higher than that of the risk score (AUC=0.695) and stage (AUC=0.779), suggesting a high credibility of the nomogram. Furthermore, the clinical response of anti-PD-1/CTLA-4 immunotherapy between high- and low-risk patients showed a significant difference. Our study successfully explored a brand-new GC pathological classification based on hypoxia pathway genes and the quantification of m6A modification patterns. Comprehensive immune analysis and validation demonstrated that hypoxia clusters were reliable, and our signature could provide a new approach for clinical decision-making and immunotherapeutic strategies for GC patients.

## INTRODUCTION

Gastric cancer (GC) is the fifth most malignant tumor worldwide (1). Greater than 1 million new cases have been identified, and most cases are already advanced at diagnosis, explaining why GC has the third highest number of cancer-related deaths (2). Based on the Lauren/WHO classification and the lymph node metastasis [tumor node metastasis (TNM)] staging of tumors, current treatments exhibit a poor correlation with the molecular pathology of cancer. Despite the development of new pathological classifications, such as The Cancer Genome Atlas (TCGA) subtypes and Asian Cancer Research Group (ACRG) subtypes, the clinical predictive value of these classification systems remains insufficient (3, 4). To identify more molecular markers that are closely related to GC progression, accurately predicting developmental trends and providing individualized treatments for patients has become a troublesome point in current relevant research fields.

With greater insight into tumor research, changes in the tumor microenvironment (TME) have drawn more attention, and hypoxia plays an important role in tumorigenesis (5). Hypoxia is one of the characteristics of the microenvironment of solid tumors and one of the greatest obstacles to cancer treatment (6–8). As a master regulator of cellular adaptation to hypoxia, hypoxia-inducible factor 1 (HIF1) has been proven to extensively regulate the expression of hypoxia genes and hypoxia adaptation–related signal transduction pathways, including EPO, VEGF, iNOS, and other genes to increase oxygen transmission and PDK-1, ALDOA, bcl-2, and other genes to reduce oxygen consumption (5, 9–12). Another feature of the TME is the change in immune cells, which contributes to maintaining a complex dynamic interaction with tumor cells (13, 14). Immunotherapy, especially programmed cell death-1 (PD-1)/PD-1 ligand 1 (PD-L1), and immune checkpoint blockade (ICB), has made remarkable achievements in recent years (15, 16). However, sustained clinical responses are only induced in a minority of cancer patients, indicating that more studies on this topic should be performed (17, 18).

As the most common RNA modification in eukaryotic cells, N6-methyladenosine (m6A) not only plays a related role in immune regulation but also plays a vital role in the occurrence and development of cancer through various processes, such as proliferation, migration, and invasion (19, 20). m6A regulators consist of three types of proteins: “writers” with methyltransferase activity, “erasers” with demethylase activity, and “readers” with m6A binding sites (20–22). Recent studies have demonstrated that the abnormal m6A modification patterns change the TME and lead to tumor progression, and hypoxia plays a potential role (23, 24). Recently, several posttranscriptional modification databases have been established such as the m6AVar and RMBase databases (25, 26), which provided important information about m6A-related variants to explore the molecular mechanisms of m6A modification for experimental biologists. Moreover, 2 powerful m6A functional analysis tools ConsRM and m6A2Target (27, 28) were also developed. However, the specific mechanisms in GC remain elusive, so a comprehensive analysis of hypoxia and m6A is urgently needed and indispensable.

In this study, we identified three hypoxia pathway subtypes in GC. By correlating hypoxia with m6A modification patterns and defining the m6A score to quantify m6A modification patterns, we ultimately established a robust signature and prognostic nomogram. This study provides information on clinicopathological characteristics and a classification system that are more in line with reality and can be used to guide clinical decision-making. In addition, this study aims to improve GC patient survival.

## MATERIALS AND METHODS

### Data Collection and Preprocessing

GC patients with survival information were retrospectively collected from the Gene Expression Omnibus (GEO, http://www.ncbi.nlm.nih.gov/geo/) and The Cancer Genome Atlas (TCGA, https://portal.gdc.cancer.gov/), and GC samples without clinical data were excluded. In total, 1,673 patients from ten cohorts were enrolled, including The Cancer Genome Atlas-Stomach Adenocarcinoma (TCGA-STAD), GSE13861, GSE26899, GSE26901, GSE57303, ACRG Cohort (GSE62254), Singapore Patient Cohort (GSE15459 and GSE34942), and GSE84437 (GSE84426 and GSE84433). The TCGA-STAD cohort (FPKM normalized) was transformed into the transcripts per kilobase million (TPM) format. For microarray cohorts, the normalized matrix files with expression data and clinical information were directly downloaded and log2 transformed. The remaining cohorts except TCGA-STAD were merged into one cohort, and the “sva” R package was employed to remove batch effects (29). The predictive value of the nomogram was tested using an additional cohort GSE28541. In addition, two immune checkpoint blockade treatment cohorts (IMvigor210 for PD-1 treatment and Nathanson2017 for CTLA-4 treatment) were obtained, and the corresponding normalized data were utilized to determine whether the m6A-related hypoxia signature could be used to screen immunotherapy-sensitive patients. Details are provided in **Supplementary Table S1**.

### Unsupervised Hierarchical Clustering Reveals Distinct Characteristics of Different Clusters

We systematically collected a set of hypoxia-related genes (https://www.gsea-msigdb.org/gsea/msigdb/cards/HALLMARK_HYPOXIA) and a total of 23 m6A regulators, including 8 writers (METTL3, METTL14, WTAP, RBM15, RBM15B, ZC3H13, CBLL1, and VIRMA), 2 erasers (ALKBH5 and FTO), and 13 readers (IGF2BP1/2/3, YTHDF1/2/3, YTHDC1/2, FMR1, ELAVL1, HNRNPC, HNRNPA2B1, and LRPPRC) (22, 30). To group hypoxia clusters, we performed principal component analysis (PCA) for data reduction. According to the Kaiser–Harris criterion, principal components <1% were considered noise and removed. After calculating the Euclidean distance, the ten-combined cohort was grouped using unsupervised hierarchical clustering with the “ward. D2” linkage criterion. Target genes for increasing oxygen delivery and reducing oxygen consumption were obtained from the Hypoxia-inducible factor 1 (HIF-1) signaling pathway to explore the hypoxic status between different clusters. Similarly, the hierarchical clustering method using the “ward.D” linkage criterion divides patients into high, medium, and low clusters according to m6A regulators. Furthermore, the results were visualized in clustering dendrograms, PCA, and t-distributed stochastic neighbor embedding (t-SNE) figures, and Kaplan Meier (KM) curves were employed to show the trends of overall survival (OS) and recurrence-free survival (RFS). Additionally, we explored the connections between the subtypes defined above and previous molecular stratifications of GC *via* a percentage stacking diagram (3, 4, 31).

### Pathway Enrichment Analysis and Single-Sample Gene Set Enrichment Analysis

Using the HALLMARK gene set (downloaded from the MSigDB database v7.1) as the background pathway, gene set variation analysis (GSVA) was performed using the “GSVA” R package to show pathway differences in 3 hypoxiaClusters (32). Moreover, based on immunity-related gene sets reported in a previously published article (33), we employed GSVA enrichment analysis to investigate the distinct response patterns of hypoxiaClusters in innate and adaptive immunity.

Single-sample gene set enrichment analysis (ssGSEA) was performed for other gene sets obtained from previously published studies as follows: the biomarkers of biological processes according to Mariathasan et al. (34), hypoxia biomarkers, T-cell dysfunction, and immunotherapy resistance biomarkers and immunosuppressive cell signatures (**Supplementary Table 2**).

### Immune Cell Infiltration Estimation

For each sample, the ESTIMATE algorithm was adopted to assess the tumor purity and population estimation of stromal and immune cells based on gene expression (35). Twenty-eight different immune cell infiltration patterns, including cells executing antitumor reactivity and cells delivering protumor suppression, were calculated from the gene sets reported in a previous study *via* ssGSEA (**Supplementary Table 2**). Furthermore, an additional 22 immune cells calculated by the CIBERSORT deconvolution algorithm, including neutrophils, eosinophils, mast cells, dendritic cells, macrophages, natural killer cells, regulatory T cells (Tregs), B cells, CD4+ T cells, CD8+ T cells, and plasma cells, were assessed to quantify infiltrating pattern heterogeneity (36).

### Quantization of the Modification Pattern of m6A Regulators

Based on the m6A score construction method of Shen et al. (22), differentially expressed genes (DEGs) between 3 m6A clusters were extracted using the “limma” R package (37). Univariate Cox regression analysis was performed on DEGs with a p-value <0.05; then, screened genes were employed for the construction of the m6A score and normalized from -1 to 1 to reduce the effect of the gene expression value. Afterwards, we calculated the hazard ratio (HR) of all screened genes and divided them into two groups based on a cut-off score of HR=1, and the m6A score was defined as the difference value of the sums in each group. Tumor mutation burden (TMB) was calculated using “maftools” according to the somatic mutation data acquired from the TCGA database (38). Pearson correlation analysis was employed to reveal the correlation between m6A and TMB. Subsequently, the distribution differences in somatic mutations between the low and high m6A score groups were analyzed and visualized using a waterfall diagram.

### Establishment of an m6A-Related Hypoxia Signature by Machine Learning

Differential expression analysis was performed to screen differentially expressed hypoxiaCluster genes between different hypoxiaClusters. The m6A-related hypoxia pathway genes (MRHPPGs) were defined based on the following criteria: correlation r >0.5 and p-value <0.001 between DEHCGs and the m6A core. Data partitioning and standardization were processed using the classification and regression training (caret) package in the R(caret) package (39). First, the entire dataset was divided into a training and testing cohort at a ratio of 6:4 using a stratified sampling method for each cohort. Then, the function “preProcess” was used to standardize the training cohort and other cohorts based on the parameters calculated in the training cohort. For feature engineering, we used 80% of the samples randomly chosen from the original sample each time. After 1,000 bootstrapping replications, genes with a p-value <0.01 that appeared greater than 900 times in univariate Cox proportional hazards regression analysis were included in further analyses. Furthermore, an algorithm integrating LASSO and bootstrapping was used to identify the best candidate predictive genes (40, 41). The optimal candidates that were repeated more than 600 times in 1,000 iterations were determined through 5 cross-validations. Then, stepwise multivariate Cox regression analysis was used to build a prognostic signature, which was determined as follows: risk score = Σ expression level of gene Xi × Cox coefficient of gene Xi. A survival decision tree was used to show the process of clinical decision-making. The predictive ability of the nomogram for 3-, 5- and 7-year OS was assessed, and external validation was performed using cohort GSE28541. A calibration curve was generated to compare the predicted survival rates with the observed survival rates (42).

### Cell Culture and Quantitative Real-Time PCR

The human gastric epithelial cell line GES-1 and GC cell lines HGC-27, NCI-N87 were obtained from Shanghai Anwei Biotechnology Co., LTD, China. GES-1 and NCI-N87 were cultured in an Roswell Park Memorial Institute (RPMI) 1640 (Gibco, US) medium, and HGC-27 was cultured in a Dulbecco's modified eagle medium (DMEM) (Gibco, US) medium with 10% fetal bovine serum (FBS; Gibco) and 1% penicillin/streptomycin in a humidified atmosphere of 5% CO_2_ and 20% O_2_ at 37°C. Total RNA was extracted using FastPure Cell/Tissue Total RNA Isolation Kit V2 (Vazyme, RC112-01) and reverse-transcribed into cDNA using HiScript III All-in-one RT SuperMix Perfect for qPCR (Vazyme, R333-01). Real-time PCR was performed using ChamQ Universal SYBR qPCR Master Mix (Vazyme, Q711-02). The primer pairs used in qRT‐PCR were as follows: APOD 5′‐AATCGAAGGTGAAGCCACCC‐3′ (forward) and 5′-GTGCCGATGGCATAAACCAG‐3′ (reverse); CCN3 5′‐AGGCAGAGTTTCAGTGCTCC‐3′ (forward) and 5′‐TGCAGGTCCCAATGACCATC‐3′ (reverse); DACT1 5′‐TTGAACTGTTTGAGGCGAAGAG‐3′ (forward) and 5′‐ACTGAACACCGAGTTAGAGGAAT‐3′ (reverse); EML1 5′‐CAGTTCTGCAACGATGACAGC‐3′ (forward) and 5′‐GCCGAACCACATCAGCTAGAG‐3′ (reverse); MMP23B 5′‐TAGGCTTCTACCCGATCAACC‐3′ (forward) and 5′‐CGCTGTCGTCGAAGTGGAT‐3′ (reverse); RBPMS2 5′‐AAGACAGCCTGTTGGTTTTGT‐3′ (forward) and 5′‐CGAATACCGTTCAGCGCATT‐3′ (reverse); TUBB6 5′‐TGGTGGACTTAGAGCCAGG‐3′ (forward) and 5′‐CCCTTTCGCCCAGTTGTTC‐3′ (reverse).

### Western Blotting

The tumor cells were lysed with RIPA buffer containing a protease inhibitor cocktail. These cells were kept on ice for approximately 1 h and vortexed every 15 min at 12,000 rpm and centrifuged for 15 min. The protein concentration in the lysate supernatant was measured by Bicinchoninic acid (BCA). The whole lysates were diluted to the same concentration, 80 μl of lysates were taken and 20 μl of 5×SDS-PAGE loading buffer were added. The samples were boiled for 15 min. Approximately 10 μl for each sample were loaded when running Sodium dodecyl sulfate-Polyacrylamide gel electrophoresis (SDS-PAGE). The protein was fractionated by 12.5% SDS-PAGE. The protein was transferred to the Polyvinylidene difluoride (PVDF) membrane at 300 V for 1 h in an ice bath. The membrane was blocked with 5% Milk-TBST for 2 h at room temperature. Then, the membrane was probed with primary Abs for glyceraldehyde-3-phosphate dehydrogenase (GAPDH) (1:10,000), and HIF-α (1:1,000) overnight at 4°C. HRP-conjugated anti-rabbit IgG (1: 20,000) was used as a secondary Ab. After secondary Ab incubation for 2 h at room temperature, the membrane was washed for 5 times with PBST and then blotted with an Enhanced chemiluminescence (ECL) solution. The blots were imaged in the dark room with an imaging machine.

### Statistical Analysis

All data processing was performed using the R 4.0.3 software. For two groups, statistical significance was estimated *via* unpaired Student’s t-tests for normally distributed variables and Wilcoxon rank-sum tests for nonnormally distributed variables. For more than two groups, one-way ANOVA tests and Kruskal–Wallis tests were used (43). The cut-off values of continuous variables, such as OS, were determined using the “survminer” R package. The area under the curve (AUC) of time-dependent receiver operating characteristic (ROC) curves was visualized by the “timeROC” R package (44), and the ROC curve of the immune checkpoint blockade therapy response was assessed using the “pROC” R package (45). Differences with p < 0.05 were considered statistically significant (*p < 0.05; **p < 0.01: ***p < 0.001: ****p < 0.0001).

## RESULTS

### The Hypoxia Status in Gastric Cancer

**Figure 1:**
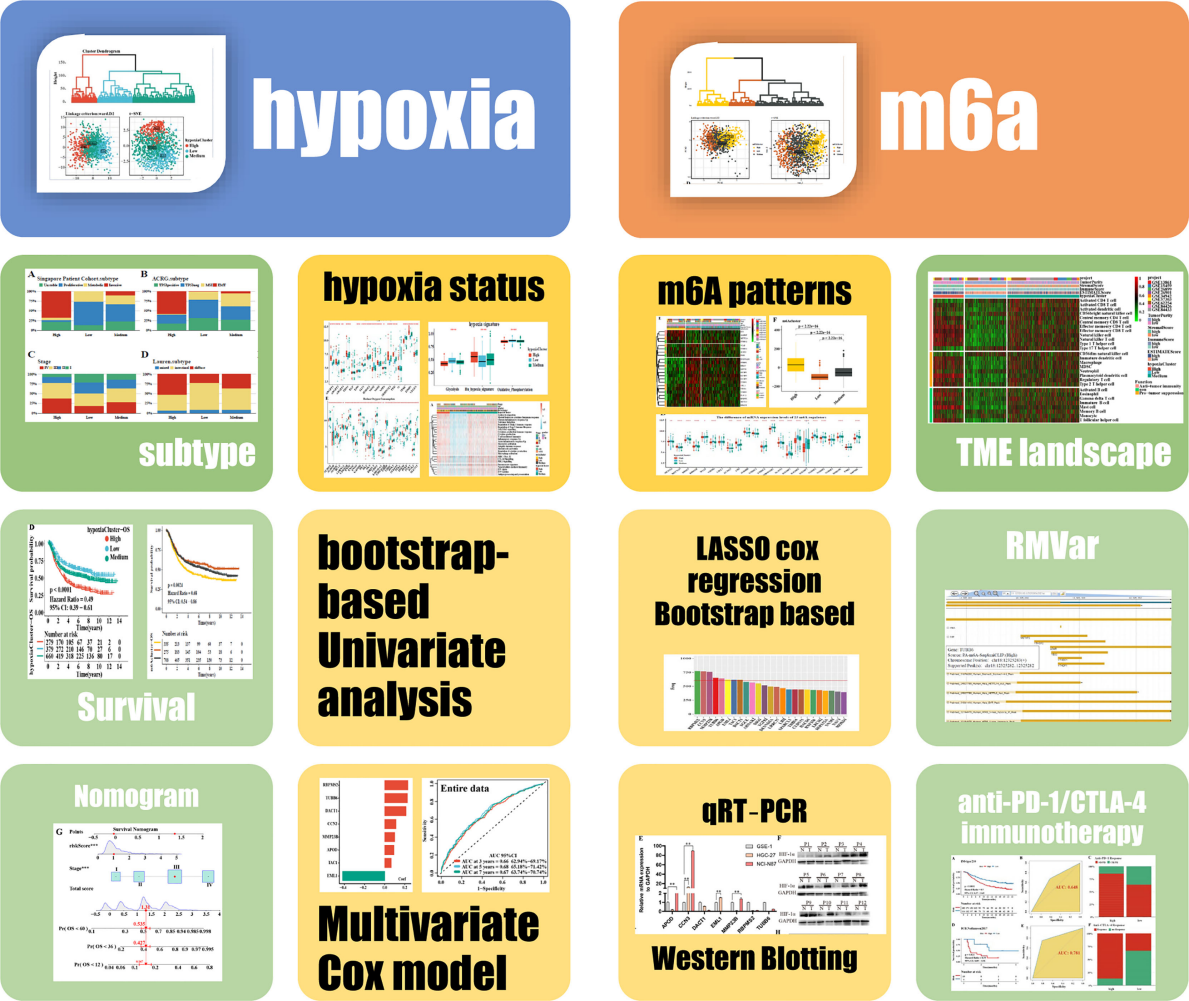
Flow chart.

**Figure 2:**
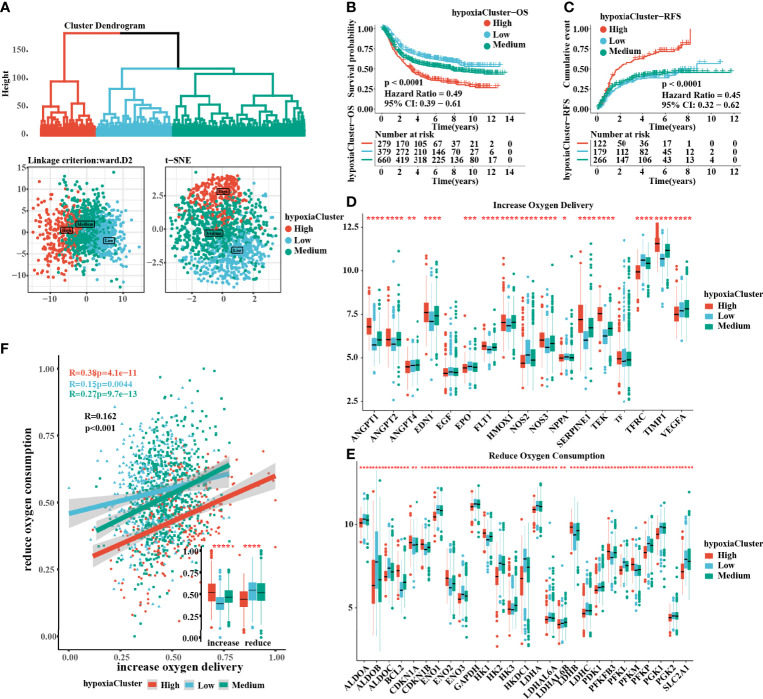
Three hypoxia types with distinct prognosis characteristic and oxygen transport status. **(A)** Identification of hypoxiaClusters by unsupervised hierarchical clustering analysis. PCA and t-SNE analysis supported to divide patients into 3 hypoxiaClusters. **(B, C)** Kaplan–Meier curves were plotted to demonstrate the difference of prognosis by OS and RFS. **(D, E)** The oxygen transport status between different hypoxiaClusters was analyzed through increasing oxygen delivery and reducing oxygen consumption target genes and correlation. The lines in the boxes represented the median value. **(F)** Between the two groups, it was revealed that cluster high had the highest increase in oxygen delivery but the lowest reduction in oxygen consumption, while the cluster low had the opposite. PCA, principal component analysis; t-SNE, t-distributed stochastic neighbor embedding; OS, overall survival; RFS, recurrence-free survival. The asterisks represented the statistical p-value (*P < 0.05; **P < 0.01; ***P < 0.001; ****p < 0.0001). The Kruskal–Wallis test was used to compare the statistical difference between three gene clusters.

The flow chart of this study is shown in (**Figure 1**). After unsupervised hierarchical clustering, we classified 3 clusters with distinct hypoxia statuses (**Figure 2A**). Next, we evaluated how the hypoxia status affected patient prognosis. Both overall survival (OS) and recurrence-free survival (RFS) prognostic analysis for the three major hypoxia statuses demonstrated a particularly prominent survival disadvantage in hypoxiaCluster-high patients **(** **Figures 2B, C**). The expression levels of target genes involved in increased oxygen delivery and reduced oxygen consumption varied among the clusters (**Figures 2D–F**), confirming that the different hypoxia clusters exhibited distinct hypoxia statuses. Patients with invasive and Epithelial-Mesenchymal Transition (EMT) subtypes were classified as hypoxiaCluster-high, whereas proliferative and TP53-negative subtypes were classified as hypoxiaCluster-low. We still observed that cancers classified as hypoxiaCluster-high exhibited poorer differentiation and were enriched in the diffuse subtype (**Supplementary Figures 1A–D**). In GC, the EMT molecular subtype and diffuse histological type were closely related to a shorter OS. Our hypoxiaCluster classification was consistent with other hypoxia characteristics (**Supplementary Figure 1E**). These results suggested that there were different hypoxia statuses with a significant prognostic value and that the GC characterized by a high hypoxia state was closely correlated with high malignancy and rapid tumor progression.

### TME Landscape in GC Tumors With Distinct Hypoxia Statuses

**Figure 3:**
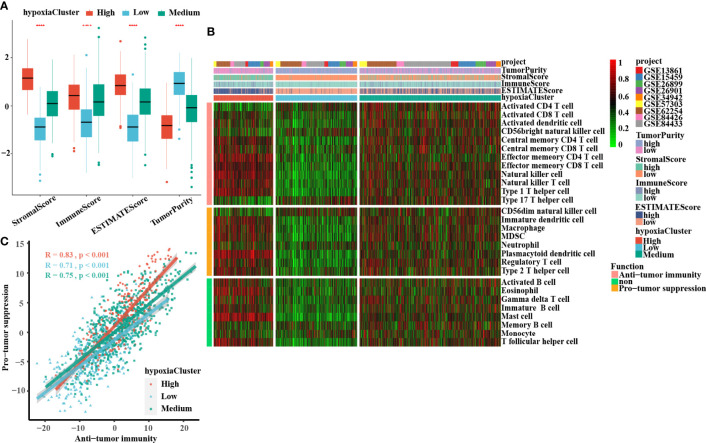
Landscape of the TME between distinct hypoxia status in GC. **(A)** The boxplots of the ESTIMATE method were used to explore the TME characteristics among these distinct hypoxia clusters, suggesting that hypoxia-cluster high had the highest stromal score, immune score, and ESTIMATE score but the lowest tumor purity (all P-values < 0.0001). The lines in the boxes represented median value. **(B)** The heatmap depicted the infiltrating difference of 28 immune cell types in 3 hypoxiaClusters. HypoxiaCluster-high had a higher abundance of immune cell infiltration, including cells performing an anti-tumor function (e.g., effector memory CD4 T cells, effector memory CD8 T cells, natural killer cells, natural killer T cells, and type 1 T helper cells) and cells executing pro-tumor suppression (e.g., immature dendritic cells, macrophages, MDSCs, neutrophils, plasmacytoid dendritic cells, regulatory T cells, and type 2 T helper cells). Moreover, hypoxia-cluster high was significantly rich in innate immunocyte infiltration including natural killer cells, macrophages, mast cells, MDSCs, and plasmacytoid dendritic cells. **(C)** The correlation between pro-tumor suppression and anti-tumor immunity was analyzed according to 3 hypoxiaClusters, respectively. Pearson’s correlation analysis suggested that the abundances of these two categories of immunocytes have a significant positive association in TME (all P-values < 0.001). The asterisks represented the statistical p-value (****p < 0.0001). The Kruskal–Wallis test was used to compare the statistical difference between three gene clusters.

A significant difference was found: hypoxiaCluster-high, which had the worst outcome, had the highest stromal score, immune score, and ESTIMATE score but had the lowest tumor purity (**Figure 3A**). Moreover, the log‐rank test revealed that patients with high stromal scores, low immune scores, high ESTIMATE scores, or low tumor purity had a poor prognosis (**Supplementary Figures 1I, J**). These results suggested that hypoxiaCluster-high might be in a stroma activation state, which is associated with a worse outcome (46). Moreover, hypoxiaCluster-high was prominently associated with high T-cell suppression and exhaustion **(** **Supplementary Figure 1F**). We conducted GSVA enrichment analysis to investigate the biological behaviors among these distinct hypoxia clusters. As shown in **Supplementary Figure 1G**, hypoxiaCluster-high was dramatically enriched in stromal and metastatic activation pathways, such as EMT, angiogenesis, myogenesis, hedgehog signaling, and TNFα signaling *via* NFκB; hypoxiaCluster-low exhibited enrichment signaling pathways associated with MYC targets V2, MYC targets V1, E2F targets, and the G2 M checkpoint. To our surprise, the subsequent analysis of infiltrating immunocyte populations suggested that hypoxiaCluster-high was significantly enriched in innate immunocytes, including natural killer cells, macrophages, mast cells, MDSCs, and plasmacytoid dendritic cells **(** **Figure 3B**; **Supplementary Figure 1H**). A previous research reported that the immune-excluded phenotype also exhibited the presence of a great number of immunocytes, but the immunocytes remained in the matrix around the nest of tumor cells rather than penetrating the parenchyma. Stromal activation in the TME is considered to promote T-cell inhibition (15). Moreover, hypoxiaCluster-high was prominently associated with high T-cell suppression and exhaustion **(** **Supplementary Figure 3B**). Thus, we conjectured that stromal activation in hypoxiaCluster-high inhibited the antitumor effect of immune cells. In addition, we found that hypoxiaCluster-high had an increased abundance of immune cell infiltration, including cells performing antitumor functions (e.g., effector memory CD4 T cells, effector memory CD8 T cells, natural killer cells, natural killer T cells, and type 1 T helper cells) and cells executing protumor suppression (e.g., immature dendritic cells, macrophages, MDSCs, neutrophils, plasmacytoid dendritic cells, regulatory T cells, and type 2 T helper cells) **(** **Figure 3B**). Pearson’s correlation analysis suggested that the abundances of these two categories of immunocytes were significantly positively associated in the TME **(** **Figure 3C**). This finding indicated the existence of a feedback mechanism in which the antitumor immune response could promote the recruitment or differentiation of cells specialized for immunosuppression. Based on the above inference, we were surprised to confirm that the three hypoxia clusters had dramatically distinct TME cell infiltration features.

### The m6A Methylation Modification Patterns Are Distinct Between Hypoxic Conditions

**Figure 4:**
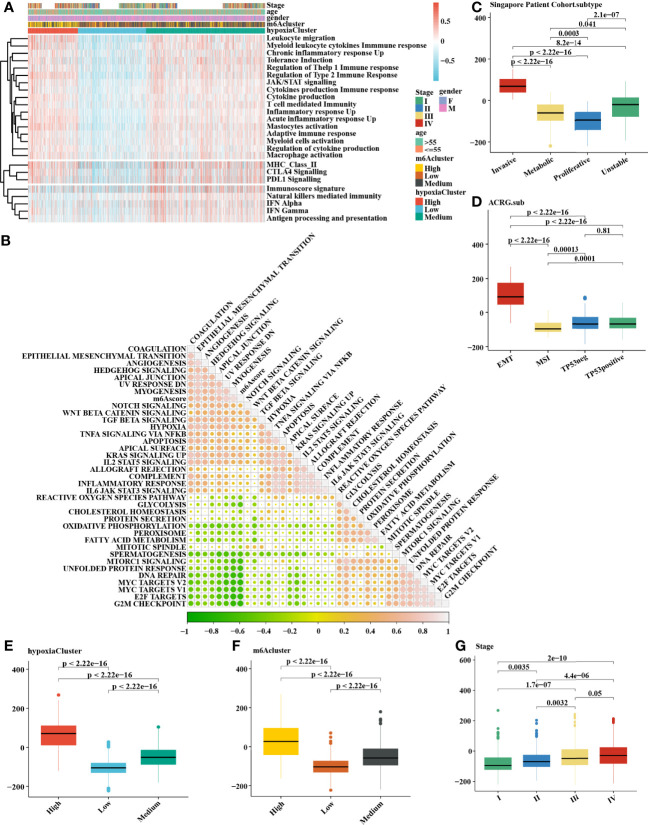
Comprehensive analysis of distinct hypoxia status between 3 m6A methylation modification patterns. **(A)** The heatmap of gene set variation analysis (GSVA) was drawn on the basis of 25 immunity-related gene sets. m6Acluster high, leukocyte migration, myeloid leukocyte cytokine immune response, and mastocyte activation were mainly concentrated in the hypoxia-cluster high, which suggested hypoxia-cluster high might be in a immunosuppressed and stromal activation state. **(B)** The correlation analysis between m6Ascore and the known signatures. Orange indicates an R value > 0, blue indicates an R value < 0. **(C–F)** Calculation of the m6Ascore in different cohorts and the correlation in different subtypes, including **(C)** Singapore patient cohort (GSE15459 and GSE34942), **(D)** ACRG cohort (GSE62254), **(E)** hypoxiaCluster, **(F)** m6Acluster, **(G)** Stage. The upper and lower ends of the boxes meant the interquartile range of values. The lines in the boxes represented the median value. MSI, microsatellite instability. The Kruskal–Wallis test was used to compare the statistical difference between three gene clusters.

It is generally accepted that m6A methylation modification is involved in diverse biological processes, including dysregulated cell death and proliferation, the degree of tumor malignancy, and immune modifications. Therefore, we similarly classified three m6A methylation modification patterns using the same analysis of hierarchical clustering mentioned above (**Supplementary Figure 2A**) based on the mRNA expression levels of 21 regulators that presented high heterogeneity (**Supplementary Figures 2D, E**). We defined these patterns as m6Acluster high, medium, and low, respectively. The Kaplan–Meier survival analysis for the three m6Aclusters demonstrated that m6Acluster low presented a remarkable survival advantage (**Supplementary Figures 2B, C**). Patients with invasive subtypes, EMT subtypes, or hypoxiaCluster-high subtypes were also mainly enriched in m6Acluster high (**Supplementary Figures 2F, H**). To further explore the biological functions affected by m6A modification phenotypes in distinct hypoxia statuses, we performed an unsupervised clustering algorithm based on hypoxia-related genes in the three m6A methylation modification patterns. Analysis indicated that patients with m6Acluster high were mainly concentrated in the hypoxiaCluster-high group **(** **Figure 4A**), which confirmed again that hypoxiaCluster-high was significantly relevant to stromal activation. To further illustrate the potential biological process associated with m6A regulator modification subtypes, we established the m6A score and further tested the relation between the known signatures and the m6A score (**Figure 4B**). We observed the distribution differences of somatic mutations between patients with high and low m6A score in the TCGA-STAD cohort. Patients with a low m6A score had more extensive TMB, and the Pearson correlation analysis confirmed that low-m6A-score tumors were significantly negatively related to tumor mutation burden (**Supplementary Figure 3A, B**). Moreover, the m6Acluster high group exhibited a significantly increased m6A score compared to the other clusters, while the m6Acluster low group showed the lowest m6A score (**Figure 4F**). In addition, patients with invasive subtypes, EMT subtypes, or IV stage had the lowest m6A score compared to other corresponding molecular/histological subtypes (**Figures 4C, D, G**), which was consistent with previous studies (46). More importantly, this is the first report that a high hypoxia status was associated with a significantly increased m6A score (**Figure 4E**). These results showed that the m6A score could also be used to evaluate certain clinical features and was closely linked to hypoxia status.

### Construction and Validation of the Prognostic Signature of m6A-Related Hypoxia Pathway Genes

**Figure 5:**
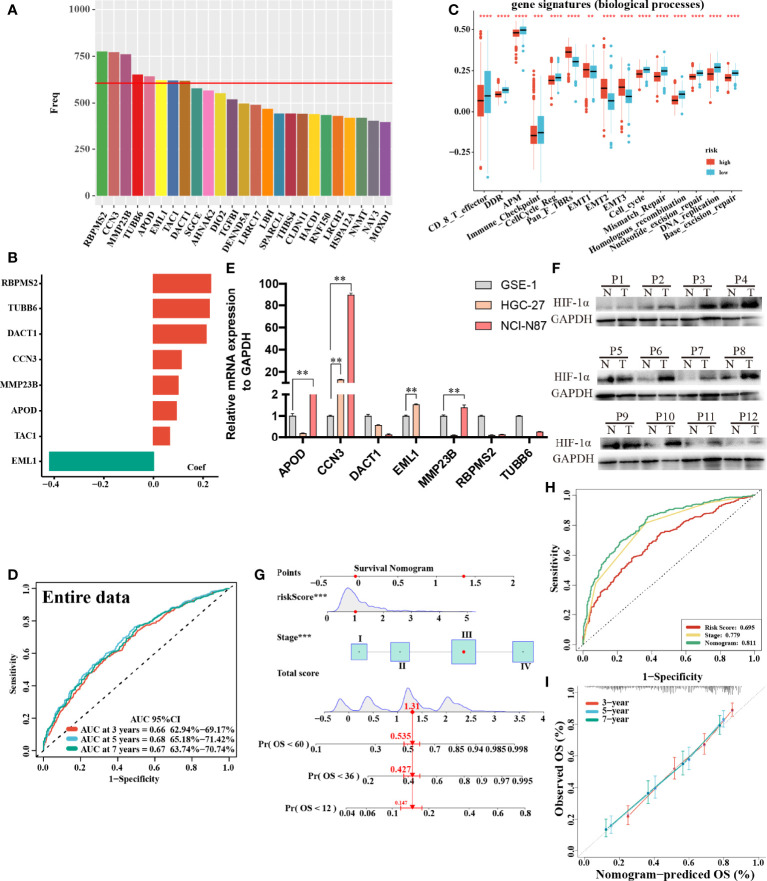
Construction and validation of signature and nomogram. **(A)** The best candidate predictive genes were selected to construct the signature according to the frequency of genes by Lasso-bootstrapping that was defined more than 600 times in 1,000 times replications. **(B)** The coefficient value of the 8 selected genes came from the stepwise multivariate Cox regression analysis. **(C)** The difference of core biological processes was evaluated between high- and low-risk patients that were divided according to the optimal cut-off value. The lines in the boxes represented the median value. **(D)** The area under the ROC curves (AUC) of the entire data were 0.66 (62.94%–69.17%, 95%CI), 0.68 (65.18%–71.42%, 95%CI), and 0.67 (63.74%–70.74%, 95%CI) at 3, 5, and 7 years, respectively. **(E)** qRT-PCR results of MRHPPG expression level in GSE-1 and GC cell lines. (Data are presented as mean ± SD., **P < 0.01). **(F)** Western blot analyses of HIF-α protein levels in total cell lysates from paired clinical specimens of normal (N) and tumor (T) tissues from 12 patients with GC. **(G)** A survival nomogram of 3-, 5-, and 7-year OS was drawn in view of the risk score and stage. **(H)** AUC for risk score, stage, and nomogram reached at 0.659, 0.779, and 0.811, respectively, which mean a high accuracy to be a reliable method. **(I)** The calibration curve of nomogram showed a favorable result in 3, 5, and 7 years. (***P < 0.001,****p < 0.0001).

**Figure 6:**
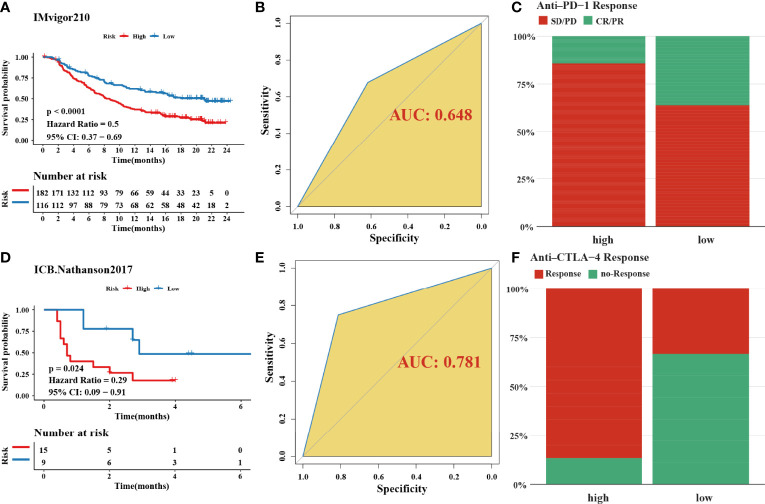
Validation of signature through two immune-checkpoint blockade treatment cohorts. **(A, D)** Kaplan–Meier curves were plotted in **(A)** IMvigor210 cohort and **(D)** Nathanson2017 cohort to confirm the credibility of the signature. **(B, E)** the AUC in IMvigor210 cohort and ICB.Nathanson2017 cohort were 0.648 and 0.781, respectively. **(C, F)** The response of immune-checkpoint blockade treatment could be connected with high- and low-risk scores, especially in anti-CTLA-4 (χ^2^ test). SD, stable disease; PD, progressive disease; CR, complete response; PR, partial response.

In total, 8 candidate predictive genes were identified (**Figures 5A, B**; **Table 1**). The formula for the risk score is as follows: risk score = 0.091592596 × APOD expression + 0.111440156 × CCN3 expression + 0.211698352 × DACT1 expression + (-0.418107247) × EML1 expression + 0.098618533 × MMP23B expression + 0.230005594 × RBPMS2 expression + 0.06395013 × TAC1 expression + 0.224582981 × TUBB6 expression. The analyses for the biological processes indicated that high-risk scores were significantly associated with increased activation of stromal pathways but presented an immunosuppressive state with decreased immune checkpoints **(** **Figure 5C**). KM curve analysis showed that patients with low risk scores had a better OS in the training cohort, which was consistent with the testing cohort, and the entire cohort served as the validation cohort **(** **Supplementary Figures 3I–K**). The prognostic accuracy of the risk score in the entire set was assessed; the areas under the ROC curve (AUCs) were 0.66 (62.94%–69.17%, 95% CI), 0.68 (65.18%–71.42%, 95% CI), and 0.67 (63.74%–70.74%, 95% CI) at 3, 5, and 7 years, respectively (**Figure 5D**). The MRHPPGs and HIF-1α expression was significantly elevated in gastric cancer. (**Figures 5E, F**) For external validation, the prognostic signature also showed a robust predictive ability (**Supplementary Figures 3C–G**). Moreover, we used the clinicopathological variables and risk score to establish a nomogram quantifying the risk assessment (**Figure 5G**). The predicted AUC values were 0.811 and 0.727 in the entire cohort and GSE28541, respectively (**Figure 5H** and **Supplementary Figure 3H**). The calibration curves presented a high credibility of the nomogram (**Figure 5I**). To better illustrate the prospect of the clinical application of the MRHPPG signature, a decision tree was used to visualize the stratification level, which displayed significant differences in survival (**Supplementary Figure 4**). Sankey diagrams clearly depicted that a high risk score was robustly related to other stratification classes with poor prognosis (**Supplementary Figure 4**). Next, we assessed the predictive value of the MRHPPG signature in the immunotherapeutic cohort. The AUC values of the IMvigor210 cohort and ICB.Nathanson2017 cohort in response to treatment were 0.648 and 0.781, respectively (**Figures 6A, B, D, E**, **Supplementary Figure 3I**). Moreover, we particularly investigated the ability of the risk score to predict the efficacy of anti-PD-L1 and anti-CTLA-4 immunotherapy, suggesting that low-risk patients showed a higher response rate to immunotherapy compared with high-risk patients (**Figures 6C, F** and **Supplementary Figure 3J**).

**Table 1:** Information of 8 m6A-related hypoxia pathway genes.

id	coef	HR	HR.95L	HR.95H	pvalue
APOD	0.091593	1.095918	0.953976	1.258979	0.195593
CCN3	0.11144	1.117887	0.992896	1.258612	0.065457
DACT1	0.211698	1.235775	1.074642	1.421068	0.002979
EML1	-0.41811	0.658292	0.550826	0.786723	4.27E-06
MMP23B	0.098619	1.103645	0.983001	1.239096	0.094981
RBPMS2	0.230006	1.258607	1.070618	1.479604	0.005325
TAC1	0.06395	1.066039	0.957504	1.186878	0.24309
TUBB6	0.224583	1.251801	1.066052	1.469914	0.006135

## DISCUSSION

GC is a common malignant tumor with a high recurrence rate (1). Despite great advances in surgery, radiation, and chemotherapy over the past decade, the outcome for advanced GC remains poor (2). The TNM cancer staging system is currently the gold standard for the assessment of the prognosis of cancer, but this system does not consider gene heterogeneity (47). Moreover, since the prognosis of patients with GC varies greatly (48), the establishment of a robust classifier to stratify patients with precise prognosis prediction and risk stratification is urgently needed at present and is essential to maximize the benefits produced by individual therapy and a timely follow-up. A large amount of convincing evidence has suggested that various malignancies are the result of complex interactions between tumor cells and nonmalignant cells in the TME, including fibroblasts, myofibroblasts, endothelial cells, and immune cells (49), which collectively contribute to the formation of a particular niche that promotes tumor growth and metastasis (3, 50, 51). In this research, using the bioinformatics analysis technology, we identified three distinct hypoxia clusters, which exhibited significantly different m6A methylation modifications and immune cell infiltration by comprehensively mining the public transcriptional data of GC. Moreover, a risk score model was constructed on the basis of 8 MRHPPGs to predict the outcome of GC patients. These findings provide a new perspective for treatment strategies to improve the prognosis and risk stratification of patients by considering the TME characteristics and transcriptomics.

As a tumor hallmark, hypoxia (reduced oxygen availability) is caused by an imbalance between increased oxygen consumption and insufficient oxygen supply, and the clinical significance of hypoxia has been widely reported in cancer therapy (52). Although the vigorous metabolism and rapid proliferation of cancer cells can stimulate the formation of a novel vasculature system that is disorderly, only a vascular system with accurate distribution in normal tissue can facilitate the delivery of oxygenated blood (53). Here, we identified three distinct hypoxia clusters with significantly different prognoses, including cluster low with the worst prognosis and cluster high with the best prognosis, using hierarchical clustering analysis based on principal components (removing principal components less than 1%). The immune-inflamed phenotype, which is also referred to as a hot tumor, exhibited abundant immunocyte infiltration in the TME (13, 15). Although the immune-excluded phenotype also exhibited the presence of a great number of immunocytes, the immunocytes remained in the matrix around the nest of tumor cells rather than penetrating the parenchyma. The matrix was restricted to the capsule of the tumor, or it penetrated the tumor itself, illustrating that the immune cells were indeed inside the tumor (54). Consistent with the above statement, we revealed that hypoxiaCluster-high presented a significant stroma activation status and was substantially enriched in carcinogenic pathways, including EMT, angiogenesis, the Kirsten rat sarcoma viral oncogene homolog (KRAS) signaling pathway, myogenesis, and TGF beta signaling pathways, which are considered T-cell suppressive. Moreover, this finding further confirmed that the hypoxia cluster high was in an obvious T-cell exhaustion state. Hence, through adequately exploring the characteristics of TME cell infiltration induced by distinct hypoxia states, it was not surprising that hypoxiaCluster-high had activated innate immunity but the poorest prognosis.

m6A methylation is the most common intracellular modification and is ubiquitously present in eukaryotic mRNA (19). Accumulating evidence supports a close link between m6A regulators and hypoxic states. A recent study reported that tumor hypoxia leads to the epigenetic remodeling of m6A (55). Qing et al. (56) reported that HIF-1α-induced YTHDF1 expression was closely related to hypoxia-induced autophagy-related HCC progression. However, the biological function of m6A methylation modification in distinct hypoxia-induced immune states remains unknown. Herein, we defined three m6A subtypes with different clinical outcomes *via* the same analysis of hierarchical clustering, which further confirmed that m6A methylation dysregulation plays a critical role in the tumorigenesis and progression of various neoplasms. Specifically, m6Acluster high comprised the worst prognosis and was related to the highest hypoxic state; m6Acluster low was associated with the best prognosis and correlated with the lowest hypoxic state. By clarifying m6A gene signatures and establishing the scoring system, we could further precisely assess the effect of m6A modification patterns on GC. Patients with invasive, EMT, m6Acluster high, and IV stage subtypes were significantly associated with a higher m6A score, which demonstrated that the m6A score was a reliable and robust tool for comprehensively evaluating m6A modification patterns and an independent prognostic biomarker for predicting patient survival in GC. Detailed associations between the m6A score and clinicopathological characteristics were found in our research. Our data also suggested a substantially negative relationship between the m6A score and tumor mutation burden (TMB). Moreover, we found that the high hypoxia cluster had a higher m6A score and that the low hypoxia cluster had a lower m6A score. Based on these results, we hypothesize that the immune-excluded phenotype of GC patients was accompanied by the activation of the m6A-related hypoxia pathway and the acquisition of other biological abilities, such as EMT and angiogenesis. Previous studies reported that EMT- and TGFβ-related signaling pathway activation led to a weakened transport of T cells into tumors as well as decreased tumor cytotoxicity (34, 57).

Finally, our study focused on the MRHPPG signature that demonstrated a prognostic value. In the training group, we initially recognized 25 MRHPPGs correlated with a prognosis and established a prognostic signature comprising 8 MRHPPGs *via* multivariate Cox regression and bootstrap-based univariate analysis with LASSO. Kaplan–Meier analysis suggested that the overall survival of patients with low risk scores was better than that of patients with high risk scores. A dramatically distinct risk score existed between nonresponders and responders, suggesting that we could more accurately predict the GC patients’ clinical response to anti-PD-1/CTLA-4 immunotherapy through the MRHPPG risk score. In addition, the analyses of the biological activity of the gene signature indicated that high risk scores were significantly related to lower CD8-positive effector T-cell activity, lower immune checkpoint responses, and higher EMT, further demonstrating that the activation of the m6A-related hypoxia pathway played an important role in immune states, especially in the immune-excluded phenotype. Next, we built a nomogram to calculate a score representing the OS of GC patients. The calibration plot suggested that the model has a satisfactory fitting curve and better clinical application than the traditional staging system.

Several limitations in this research should be noted. First, several independent external validations were conducted in our research, but it was still difficult to include all of the diverse features of patients from different geographic regions when cases and materials were gathered retrospectively from public databases. Second, the microenvironment features of distinct tumor spatial regions might be different; however, the samples used for analysis were all from the tumor core. Additionally, our study was not completed enough to cover related bioinformatics analysis focusing on m6A RNA modification (e.g., databases like m6AVar and RMBase and functional tools like ConsRM and m6A2Target) (25, 28, 58). Therefore, further investigations based on well-designed, prospective, multicenter studies are required.

## Data Availability Statement

The datasets presented in this study can be found in online repositories. The names of the repository/repositories and accession number(s) can be found in the article/**Supplementary Material**.

## Ethics Statement

The studies involving human participants were reviewed and approved by the Medical Ethics Committee of the Second Affiliated Hospital of Nanchang University. The patients/participants provided their written informed consent to participate in this study.

## Author Contributions

ZZ, HL and Z-KN conceived and designed this study. Z-KN and H-KT collected and assembled the data. C-GH and JL drafted the manuscript. Z-LY and H-NZ revised the manuscript. All authors read and approved the final version of the manuscript.

## Funding

This study was supported by the National Natural Science Foundation of China (Grant Number: 81860433 and 82103645), Training Plan for Academic and Technical Young Leaders of Major Disciplines in Jiangxi Province (Grant Number: 20204BCJ23021), the Natural Science Youth Foundation of Jiangxi Province (Grant Numbers: 20192BAB215036), the Key Technology Research and Development Program of Jiangxi Province (Grant Number: 20202BBG73024), the Foundation for Fostering Young Scholar of Nanchang University (Grant Number: PY201822) and Science and Technology plan of Jiangxi Provincial Health and Family Planning Commission (Grant Number: 20195072) and Chinese Medicine Foundation of Jiangxi Provincial Health Commission Administration (Grant Number: 2018B038).

## Conflict of Interest

The authors declare that the research was conducted in the absence of any commercial or financial relationships that could be construed as a potential conflict of interest.

## Publisher’s Note

All claims expressed in this article are solely those of the authors and do not necessarily represent those of their affiliated organizations, or those of the publisher, the editors and the reviewers. Any product that may be evaluated in this article, or claim that may be made by its manufacturer, is not guaranteed or endorsed by the publisher.

## Supplementary Material

Supplementary Figure 1 The characteristics of 3 hypoxiaClusters. **(A–D)** The different proportion of **(A)** Singapore patient cohort subtype, **(B)** ACRG subtype, **(C)** stage and **(D)** Lauren subtype were calculated *via* 3 hypoxiaClusters, respectively. **(E)** The comparison of hypoxiaCluster and previously hypoxia clustering characteristics. **(F)** A boxplot of cell composition in different hypoxiaCluster indicated hypoxiaCluster high was associated with high T-cell suppressive and exhaustion. **(G)** The heatmap was performed to reveal the difference of gene enrichment in 3 hypoxiaClusters. **(H)** The result of CIBERSORT deconvolution algorithm was described to assess the immune cell composition in 3 hypoxiaClusters. Only red name with “*” means statistical significance. **(I–J)** Kaplan–Meier curves to display prognostic difference after dividing patients into high and low groups. The log‐rank test revealed that patients with high stromal score, low immune scores, high ESTIMATE score, or low tumor purity related to poor prognosis.

Supplementary Figure 2 Hierarchical clustering of m6A methylation modification regulators. **(A)** Unsupervised hierarchical clustering analysis for m6A regulators with “ward.D2” linkage criterion was exhibited by cluster dendrogram, PCA and t-SNE. **(B, C)** Kaplan–Meier curves were plotted to demonstrate the difference of prognosis by overall survival (OS) and recurrence-free survival (RFS). **(D)** The difference of mRNA expression level of 23 m6A regulators was plotted in a boxplot. **(E)** the heatmap was drawn to display the relationship of 23 m6A regulators and m6A clusters. **(F–H)** The different subtypes proportion of (F) Singapore patient cohort subtype, **(G)** ACRG subtype and (H) hypoxiaCluster showed specific connection with m6A clusters.

Supplementary Figure 3 Further analysis on m6Ascore and validation of the signature and nomogram. **(A)**.Relationship between m6Ascore and tumor microenvironment burden (TMB) was plotted after student-t test, and r_Person_ was -0.50 and CI_95%_ [-0.58, -0.42]. **(B)** The waterfall plot depicted tumor somatic mutation with low m6Ascore. The numbers and bar plot on the right showed the mutation frequency of each gene and the proportion of each variant type, respectively. **(C)** Kaplan–Meier curves of TCGA cohort to be for validation. **(D)** The AUC in TCGA-STAD cohort achieved 0.60 (53.66%-66.89%, 95%CI), 0.65 (57.03%-72.31%, 95%CI), and 0.61 (47.81%-73.75%, 95%CI) at 3, 5, and 7 years respectively. **(E)** Different proportions of TCGA cohort subtype were influenced by high- and low-risk score. **(F)** An external validation cohort of GSE28541 showed significant difference of m6A. **(G)** the AUC 95% CI were 0.66 (57.27% - 74.76%, 95%CI), 0.64 (56.35% - 72.46%, 95%CI), and 0.64 (55.95% - 71.49%, 95%CI) at 3, 5, and 7 years respectively. **(H)** AUC for riskscore, stage and nomogram attained 0.646, 0.704 and 0.727, respectively, according to the m6Ascore. **(I–K)** Kaplan–Meier curves to show the OS difference were depicted on training cohort, testing cohort, and the entire cohort (P < 0.0001, log-rank test).

Supplementary Figure 4 Decision tree. **(A)** Simulating clinical decision of ACRG cohort subtypes. **(B)** Alluvial diagram was performed on hypoxiaCluster, m6Ascore, ACRG subtypes, decision cluster result, and risk clusters to analyze the mutual connection. **(C)** Kaplan–Meier curves for the decision cluster result from the ACRG decision tree. **(D)** Simulating clinical decision of Singapore patient cohort subtypes. **(E)** Alluvial diagram was performed on hypoxiaCluster, m6Ascore, Singapore patient, decision cluster result, and risk clusters to analyze the mutual connection. **(F)** Kaplan–Meier curves for decision cluster result from Singapore patient decision tree.

## References

[1] Sung H Ferlay J Siegel RL Laversanne M Soerjomataram I Jemal A. Global Cancer Statistics 2020: GLOBOCAN Estimates of Incidence and Mortality Worldwide for 36 Cancers in 185 Countries. CA Cancer J Clin (2021) 71(3):209–49. doi: 10.3322/caac.21660 33538338

[2] Sexton RE Al Hallak MN Diab M Azmi AS. Gastric Cancer: A Comprehensive Review of Current and Future Treatment Strategies. Cancer Metastasis Rev (2020) 39(4):1179–203. doi: 10.1007/s10555-020-09925-3 PMC7680370 32894370

[3] Adam JB Vesteinn T Ilya S Sheila MR Michael M Brady B. Comprehensive Molecular Characterization of Gastric Adenocarcinoma. Nature (2014) 513(7517):202–9. doi: 10.1038/nature13480 PMC4170219 25079317

[4] Cristescu R Lee J Nebozhyn M Kim KM Ting JC Wong SS. Molecular Analysis of Gastric Cancer Identifies Subtypes Associated With Distinct Clinical Outcomes. Nat Med (2015) 21(5):449–56. doi: 10.1038/nm.3850 25894828

[5] Choudhry H Harris AL. Advances in Hypoxia-Inducible Factor Biology. Cell Metab (2018) 27(2):281–98. doi: 10.1016/j.cmet.2017.10.005 29129785

[6] Schito L Semenza GL. Hypoxia-Inducible Factors: Master Regulators of Cancer Progression. Trends Cancer (2016) 2(12):758–70. doi: 10.1016/j.trecan.2016.10.016 28741521

[7] Wigerup C Påhlman S Bexell D. Therapeutic Targeting of Hypoxia and Hypoxia-Inducible Factors in Cancer. Pharmacol Ther (2016) 164:152–69. doi: 10.1016/j.pharmthera.2016.04.009 27139518

[8] Yuan S Xiang Y Wang G Zhou M Meng G Liu Q. Hypoxia-Sensitive LINC01436 Is Regulated by E2F6 and Acts as an Oncogene by Targeting miR-30a-3p in non-Small Cell Lung Cancer. Mol Oncol (2019) 13(4):840–56. doi: 10.1002/1878-0261.12437 PMC6441908 30614188

[9] Peña-Mercado E Garcia-Lorenzana M Arechaga-Ocampo E González-De la Rosa CH Beltran NE. Evaluation of HIF-1α and iNOS in Ischemia/Reperfusion Gastric Model: Bioimpedance, Histological and Immunohistochemical Analyses. Histol Histopathol (2018) 33(8):815–23. doi: 10.14670/hh-11-975 29451295

[10] Chang YC Chan YC Chang WM Lin YF Yang CJ Su CY. Feedback Regulation of ALDOA Activates the HIF-1α/MMP9 Axis to Promote Lung Cancer Progression. Cancer Lett (2017) 403:28–36. doi: 10.1016/j.canlet.2017.06.001 28610954

[11] Liu T Jin L Chen M Zheng Z Lu W Fan W. Ku80 Promotes Melanoma Growth and Regulates Antitumor Effect of Melatonin by Targeting HIF1-α Dependent PDK-1 Signaling Pathway. Redox Biol (2019) 25:101197. doi: 10.1016/j.redox.2019.101197 31023624 PMC6859552

[12] Niu Y Lin Z Wan A Sun L Yan S Liang H. Loss-Of-Function Genetic Screening Identifies ALDOA as an Essential Driver for Liver Cancer Cell Growth Under Hypoxia. Hepatology (2021) 74(3):1461–79. doi: 10.1002/hep.31846 PMC8518375 33813748

[13] Turley SJ Cremasco V Astarita JL. Immunological Hallmarks of Stromal Cells in the Tumour Microenvironment. Nat Rev Immunol (2015) 15(11):669–82. doi: 10.1038/nri3902 26471778

[14] Kather JN Suarez-Carmona M Charoentong P Weis CA Hirsch D Bankhead P. Topography of Cancer-Associated Immune Cells in Human Solid Tumors. Elife (2018) 7:e36967. doi: 10.7554/eLife.36967 PMC6133554 30179157

[15] Chen DS Mellman I. Elements of Cancer Immunity and the Cancer-Immune Set Point. Nature (2017) 541(7637):321–30. doi: 10.1038/nature21349 28102259

[16] Gong J Chehrazi-Raffle A Reddi S Salgia R. Development of PD-1 and PD-L1 Inhibitors as a Form of Cancer Immunotherapy: A Comprehensive Review of Registration Trials and Future Considerations. J Immunother Cancer (2018) 6(1):8. doi: 10.1186/s40425-018-0316-z 29357948 PMC5778665

[17] Routy B Le Chatelier E Derosa L Duong CPM Alou MT Daillère R. Gut Microbiome Influences Efficacy of PD-1-Based Immunotherapy Against Epithelial Tumors. Science (2018) 359(6371):91–7. doi: 10.1126/science.aan3706 29097494

[18] Gibney GT Weiner LM Atkins MB. Predictive Biomarkers for Checkpoint Inhibitor-Based Immunotherapy. Lancet Oncol (2016) 17(12):e542–e51. doi: 10.1016/S1470-2045(16)30406-5 PMC5702534 27924752

[19] Wang X Zhao BS Roundtree IA Lu Z Han D Ma H. N(6)-Methyladenosine Modulates Messenger RNA Translation Efficiency. Cell (2015) 161(6):1388–99. doi: 10.1016/j.cell.2015.05.014 PMC4825696 26046440

[20] Ma S Chen C Ji X Liu J Zhou Q Wang G. The Interplay Between M6a RNA Methylation and Noncoding RNA in Cancer. J Hematol Oncol (2019) 12(1):121. doi: 10.1186/s13045-019-0805-7 31757221 PMC6874823

[21] He L Li H Wu A Peng Y Shu G Yin G. Functions of N6-Methyladenosine and Its Role in Cancer. Mol Cancer (2019) 18(1):176. doi: 10.1186/s12943-019-1109-9 31801551 PMC6892141

[22] Shen X Hu B Xu J Qin W Fu Y Wang S. The M6a Methylation Landscape Stratifies Hepatocellular Carcinoma Into 3 Subtypes With Distinct Metabolic Characteristics. Cancer Biol Med (2020) 17(4):937–52. doi: 10.20892/j.issn.2095-3941.2020.0402 PMC7721089 33299645

[23] Wang YJ Yang B Lai Q Shi JF Peng JY Zhang Y. Reprogramming of M(6)A Epitranscriptome Is Crucial for Shaping of Transcriptome and Proteome in Response to Hypoxia. RNA Biol (2021) 18(1):131–43. doi: 10.1080/15476286.2020.1804697 PMC7834094 32746693

[24] Gu Y Wu X Zhang J Fang Y Pan Y Shu Y. The Evolving Landscape of N(6)-Methyladenosine Modification in the Tumor Microenvironment. Mol Ther (2021) 29(5):1703–15. doi: 10.1016/j.ymthe.2021.04.009 PMC8116604 33839323

[25] Zheng Y Nie P Peng D He Z Liu M Xie Y. M6avar: A Database of Functional Variants Involved in M6a Modification. Nucleic Acids Res (2018) 46(D1):D139–D45. doi: 10.1093/nar/gkx895 PMC5753261 29036329

[26] Xuan JJ Sun WJ Lin PH Zhou KR Liu S Zheng LL. RMBase V2.0: Deciphering the Map of RNA Modifications From Epitranscriptome Sequencing Data. Nucleic Acids Res (2018) 46(D1):D327–D34. doi: 10.1093/nar/gkx934 PMC5753293 29040692

[27] Song B Chen K Tang Y Wei Z Su J de Magalhaes JP. ConsRM: Collection and Large-Scale Prediction of the Evolutionarily Conserved RNA Methylation Sites, With Implications for the Functional Epitranscriptome. Brief Bioinform (2021) 22(6):bbab088. doi: 10.1093/bib/bbab088 33993206

[28] Deng S Zhang H Zhu K Li X Ye Y Li R. M6A2Target: A Comprehensive Database for Targets of M6a Writers, Erasers and Readers. Brief Bioinform (2021) 22(3):bbaa055. doi: 10.1093/bib/bbaa055 32392583

[29] Leek JT Johnson WE Parker HS Jaffe AE Storey JD. The Sva Package for Removing Batch Effects and Other Unwanted Variation in High-Throughput Experiments. Bioinformatics (2012) 28(6):882–3. doi: 10.1093/bioinformatics/bts034 PMC3307112 22257669

[30] Liu ZX Li LM Sun HL Liu SM. Link Between M6a Modification and Cancers. Front Bioeng Biotechnol (2018) 6:89. doi: 10.3389/fbioe.2018.00089 30062093 PMC6055048

[31] Ooi CH Ivanova T Wu J Lee M Tan IB Tao J. Oncogenic Pathway Combinations Predict Clinical Prognosis in Gastric Cancer. PloS Genet (2009) 5(10):e1000676. doi: 10.1371/journal.pgen.1000676 19798449 PMC2748685

[32] Hänzelmann S Castelo R Guinney J. GSVA: Gene Set Variation Analysis for Microarray and RNA-Seq Data. BMC Bioinf (2013) 14:7. doi: 10.1186/1471-2105-14-7 PMC3618321 23323831

[33] García-Mulero S Alonso MH Pardo J Santos C Sanjuan X Salazar R. Lung Metastases Share Common Immune Features Regardless of Primary Tumor Origin. J Immunother Cancer (2020) 8(1):e000491. doi: 10.1136/jitc-2019-000491 PMC7319789 32591432

[34] Mariathasan S Turley SJ Nickles D Castiglioni A Yuen K Wang Y. Tgfβ Attenuates Tumour Response to PD-L1 Blockade by Contributing to Exclusion of T Cells. Nature (2018) 554(7693):544–8. doi: 10.1038/nature25501 PMC6028240 29443960

[35] Yoshihara K Shahmoradgoli M Martínez E Vegesna R Kim H Torres-Garcia W. Inferring Tumour Purity and Stromal and Immune Cell Admixture From Expression Data. Nat Commun (2013) 4:2612. doi: 10.1038/ncomms3612 24113773 PMC3826632

[36] Newman AM Liu CL Green MR Gentles AJ Feng W Xu Y. Robust Enumeration of Cell Subsets From Tissue Expression Profiles. Nat Methods (2015) 12(5):453–7. doi: 10.1038/nmeth.3337 PMC4739640 25822800

[37] Ritchie ME Phipson B Wu D Hu Y Law CW Shi W. Limma Powers Differential Expression Analyses for RNA-Sequencing and Microarray Studies. Nucleic Acids Res (2015) 43(7):e47. doi: 10.1093/nar/gkv007 25605792 PMC4402510

[38] Mayakonda A Lin DC Assenov Y Plass C Koeffler HP. Maftools: Efficient and Comprehensive Analysis of Somatic Variants in Cancer. Genome Res (2018) 28(11):1747–56. doi: 10.1101/gr.239244.118 PMC6211645 30341162

[39] Kerker M. Classics and Classicists of Colloid and Interface Science 8. Albert Einstein. J Colloid Interface Sci (1989) 129(1):291–95. doi: 10.1016/0021-9797(89)90442-6

[40] Park T Casella G. The Bayesian Lasso. J Am Stat Assoc (2008) 103(482):681–6. doi: 10.1198/016214508000000337

[41] Zeng D Ye Z Wu J Zhou R Fan X Wang G. Macrophage Correlates With Immunophenotype and Predicts Anti-PD-L1 Response of Urothelial Cancer. Theranostics (2020) 10(15):7002–14. doi: 10.7150/thno.46176 PMC7295060 32550918

[42] Austin PC Steyerberg EW. Graphical Assessment of Internal and External Calibration of Logistic Regression Models by Using Loess Smoothers. Stat Med (2014) 33(3):517–35. doi: 10.1002/sim.5941 PMC4793659 24002997

[43] Hazra A Gogtay N. Biostatistics Series Module 3: Comparing Groups: Numerical Variables. Indian J Dermatol (2016) 61(3):251–60. doi: 10.4103/0019-5154.182416 PMC4885176 27293244

[44] Blanche P Dartigues JF Jacqmin-Gadda H. Estimating and Comparing Time-Dependent Areas Under Receiver Operating Characteristic Curves for Censored Event Times With Competing Risks. Stat Med (2013) 32(30):5381–97. doi: 10.1002/sim.5958 24027076

[45] Robin X Turck N Hainard A Tiberti N Lisacek F Sanchez JC. pROC: An Open-Source Package for R and S+ to Analyze and Compare ROC Curves. BMC Bioinf (2011) 12:77. doi: 10.1186/1471-2105-12-77 PMC3068975 21414208

[46] Zhang B Wu Q Li B Wang D Wang L Zhou YL. M(6)A Regulator-Mediated Methylation Modification Patterns and Tumor Microenvironment Infiltration Characterization in Gastric Cancer. Mol Cancer (2020) 19(1):53. doi: 10.1186/s12943-020-01170-0 32164750 PMC7066851

[47] Gao JP Xu W Liu WT Yan M Zhu ZG. Tumor Heterogeneity of Gastric Cancer: From the Perspective of Tumor-Initiating Cell. World J Gastroenterol (2018) 24(24):2567–81. doi: 10.3748/wjg.v24.i24.2567 PMC6021770 29962814

[48] Liu Y Wu J Huang W Weng S Wang B Chen Y. Development and Validation of a Hypoxia-Immune-Based Microenvironment Gene Signature for Risk Stratification in Gastric Cancer. J Transl Med (2020) 18(1):201. doi: 10.1186/s12967-020-02366-0 32410620 PMC7226948

[49] Allen M Louise Jones J. Jekyll and Hyde: The Role of the Microenvironment on the Progression of Cancer. J Pathol (2011) 223(2):162–76. doi: 10.1002/path.2803 21125673

[50] Jiang Y Xie J Huang W Chen H Xi S Han Z. Tumor Immune Microenvironment and Chemosensitivity Signature for Predicting Response to Chemotherapy in Gastric Cancer. Cancer Immunol Res (2019) 7(12):2065–73. doi: 10.1158/2326-6066.CIR-19-0311 31615816

[51] Lin R Zhang H Yuan Y He Q Zhou J Li S. Fatty Acid Oxidation Controls CD8(+) Tissue-Resident Memory T-Cell Survival in Gastric Adenocarcinoma. Cancer Immunol Res (2020) 8(4):479–92. doi: 10.1158/2326-6066.CIR-19-0702 32075801

[52] Harris AL. Hypoxia–a Key Regulatory Factor in Tumour Growth. Nat Rev Cancer (2002) 2(1):38–47. doi: 10.1038/nrc704 11902584

[53] Jing X Yang F Shao C Wei K Xie M Shen H. Role of Hypoxia in Cancer Therapy by Regulating the Tumor Microenvironment. Mol Cancer (2019) 18(1):157. doi: 10.1186/s12943-019-1089-9 31711497 PMC6844052

[54] Joyce JA Fearon DT. T Cell Exclusion, Immune Privilege, and the Tumor Microenvironment. Science (2015) 348(6230):74–80. doi: 10.1126/science.aaa6204 25838376

[55] Gu C Wang Z Zhou N Li G Kou Y Luo Y. Mettl14 Inhibits Bladder TIC Self-Renewal and Bladder Tumorigenesis Through N(6)-Methyladenosine of Notch1. Mol Cancer (2019) 18(1):168. doi: 10.1186/s12943-019-1084-1 31760940 PMC6876123

[56] Li Q Ni Y Zhang L Jiang R Xu J Yang H. HIF-1α-Induced Expression of M6a Reader YTHDF1 Drives Hypoxia-Induced Autophagy and Malignancy of Hepatocellular Carcinoma by Promoting ATG2A and ATG14 Translation. Signal Transduct Target Ther (2021) 6(1):76. doi: 10.1038/s41392-020-00453-8 33619246 PMC7900110

[57] Tauriello DVF Palomo-Ponce S Stork D Berenguer-Llergo A Badia-Ramentol J Iglesias M. Tgfβ Drives Immune Evasion in Genetically Reconstituted Colon Cancer Metastasis. Nature (2018) 554(7693):538–43. doi: 10.1038/nature25492 29443964

[58] Luo X Li H Liang J Zhao Q Xie Y Ren J. RMVar: An Updated Database of Functional Variants Involved in RNA Modifications. Nucleic Acids Res (2021) 49(D1):D1405–D12. doi: 10.1093/nar/gkaa811 PMC7779057 33021671

