# Supplementary figures and images for: The Hypoxic Landscape Stratifies Gastric Cancer Into 3 Subtypes With Distinct M6a Methylation and Tumor Microenvironment Infiltration Characteristics
Source: Front Immunol. 2022 Jun 21;13:860041. doi: 10.3389/fimmu.2022.860041 (PMC9253390; doi:10.3389/fimmu.2022.860041)

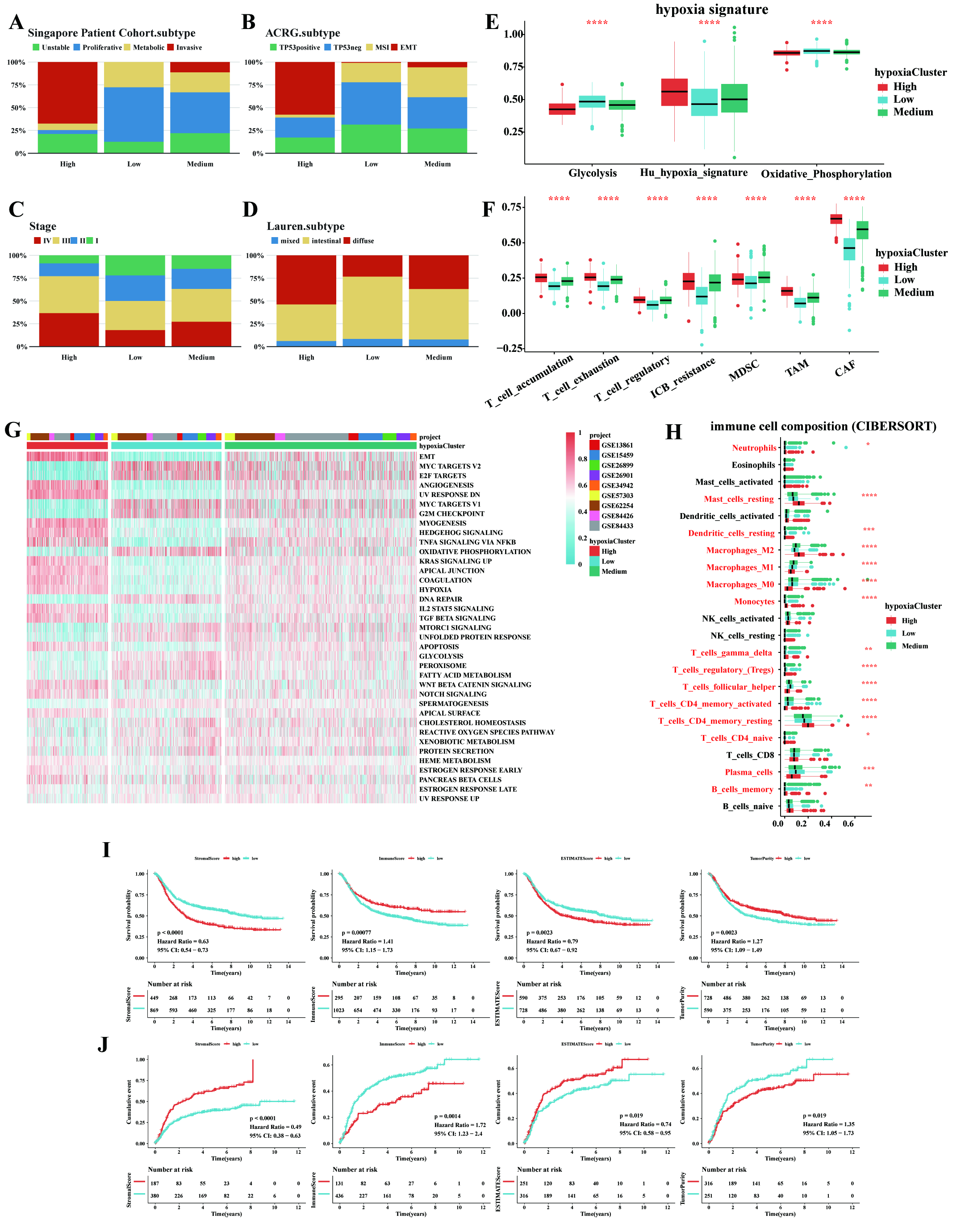

Supplement: Supplementary Figure 1 — The characteristics of 3 hypoxiaClusters. (A–D) The different proportion of (A) Singapore patient cohort subtype, (B) ACRG subtype, (C) stage and (D) Lauren subtype were calculated via 3 hypoxiaClusters, respectively. (E) The comparison of hypoxiaCluster and previously hypoxia clustering characteristics. (F) A boxplot of cell composition in different hypoxiaCluster indicated hypoxiaCluster high was associated with high T-cell suppressive and exhaustion. (G) The heatmap was performed to reveal the difference of gene enrichment in 3 hypoxiaClusters. (H) The result of CIBERSORT deconvolution algorithm was described to assess the immune cell composition in 3 hypoxiaClusters. Only red name with “*” means statistical significance. (I–J) Kaplan–Meier curves to display prognostic difference after dividing patients into high and low groups. The log‐rank test revealed that patients with high stromal score, low immune scores, high ESTIMATE score, or low tumor purity related to poor prognosis. [file Image_1.tif]

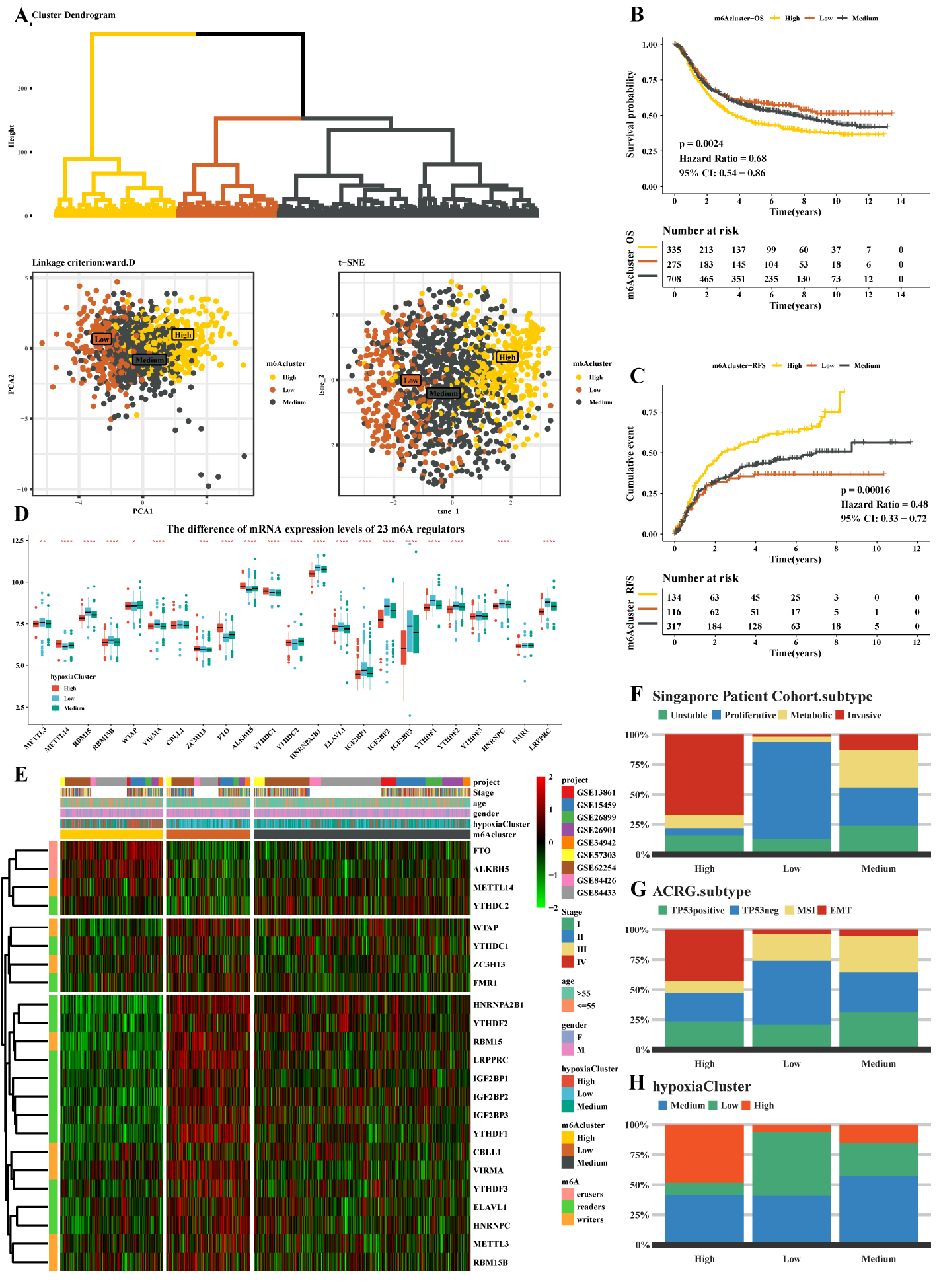

Supplement: Supplementary Figure 2 — Hierarchical clustering of m6A methylation modification regulators. (A) Unsupervised hierarchical clustering analysis for m6A regulators with “ward.D2” linkage criterion was exhibited by cluster dendrogram, PCA and t-SNE. (B, C) Kaplan–Meier curves were plotted to demonstrate the difference of prognosis by overall survival (OS) and recurrence-free survival (RFS). (D) The difference of mRNA expression level of 23 m6A regulators was plotted in a boxplot. (E) the heatmap was drawn to display the relationship of 23 m6A regulators and m6A clusters. (F–H) The different subtypes proportion of (F) Singapore patient cohort subtype, (G) ACRG subtype and (H) hypoxiaCluster showed specific connection with m6A clusters. [file Image_2.tif]

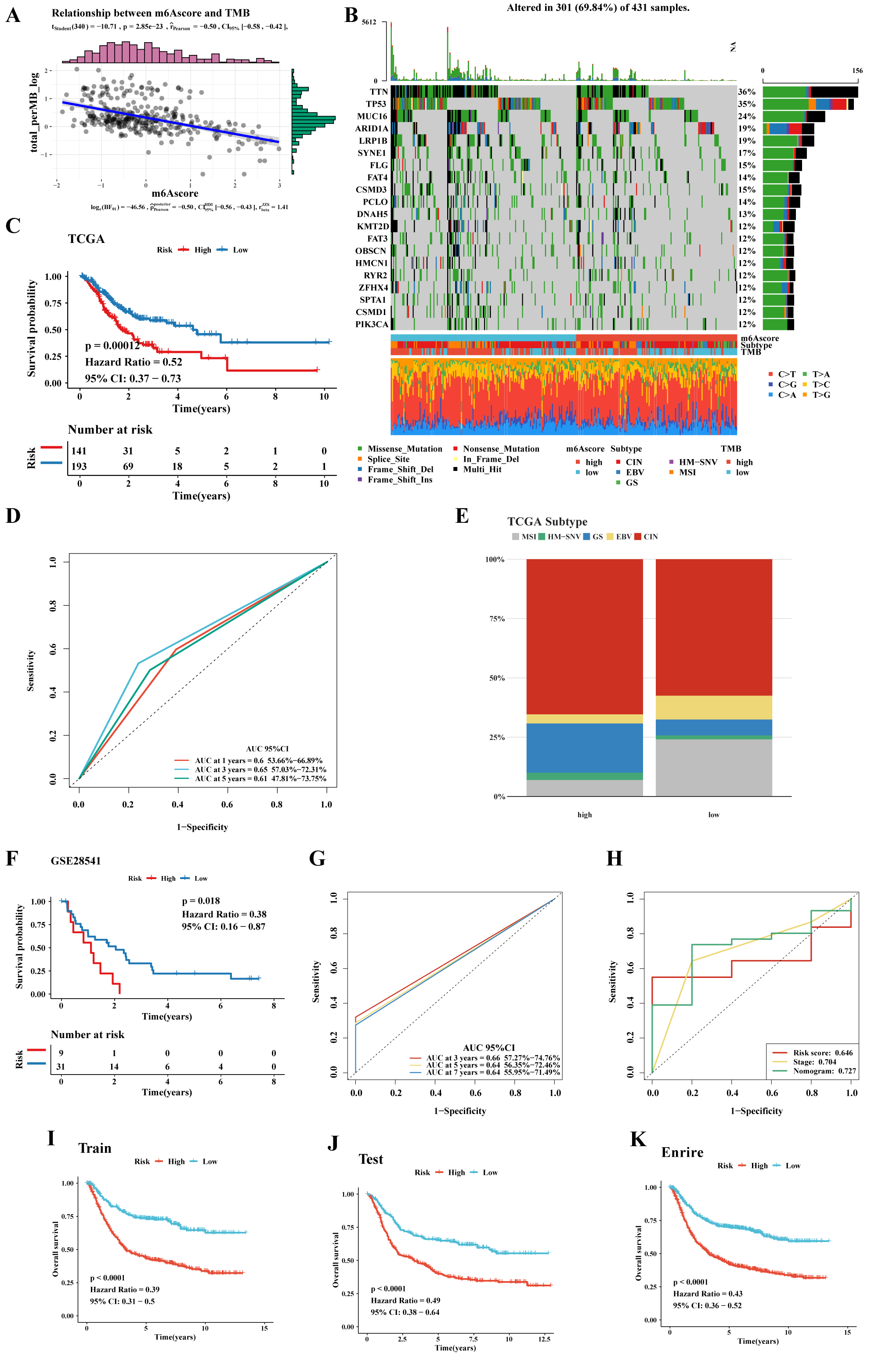

Supplement: Supplementary Figure 3 — Further analysis on m6Ascore and validation of the signature and nomogram. (A).Relationship between m6Ascore and tumor microenvironment burden (TMB) was plotted after student-t test, and rPerson was -0.50 and CI95% [-0.58, -0.42]. (B) The waterfall plot depicted tumor somatic mutation with low m6Ascore. The numbers and bar plot on the right showed the mutation frequency of each gene and the proportion of each variant type, respectively. (C) Kaplan–Meier curves of TCGA cohort to be for validation. (D) The AUC in TCGA-STAD cohort achieved 0.60 (53.66%-66.89%, 95%CI), 0.65 (57.03%-72.31%, 95%CI), and 0.61 (47.81%-73.75%, 95%CI) at 3, 5, and 7 years respectively. (E) Different proportions of TCGA cohort subtype were influenced by high- and low-risk score. (F) An external validation cohort of GSE28541 showed significant difference of m6A. (G) the AUC 95% CI were 0.66 (57.27% - 74.76%, 95%CI), 0.64 (56.35% - 72.46%, 95%CI), and 0.64 (55.95% - 71.49%, 95%CI) at 3, 5, and 7 years respectively. (H) AUC for riskscore, stage and nomogram attained 0.646, 0.704 and 0.727, respectively, according to the m6Ascore. (I–K) Kaplan–Meier curves to show the OS difference were depicted on training cohort, testing cohort, and the entire cohort (P < 0.0001, log-rank test). [file Image_3.tif]

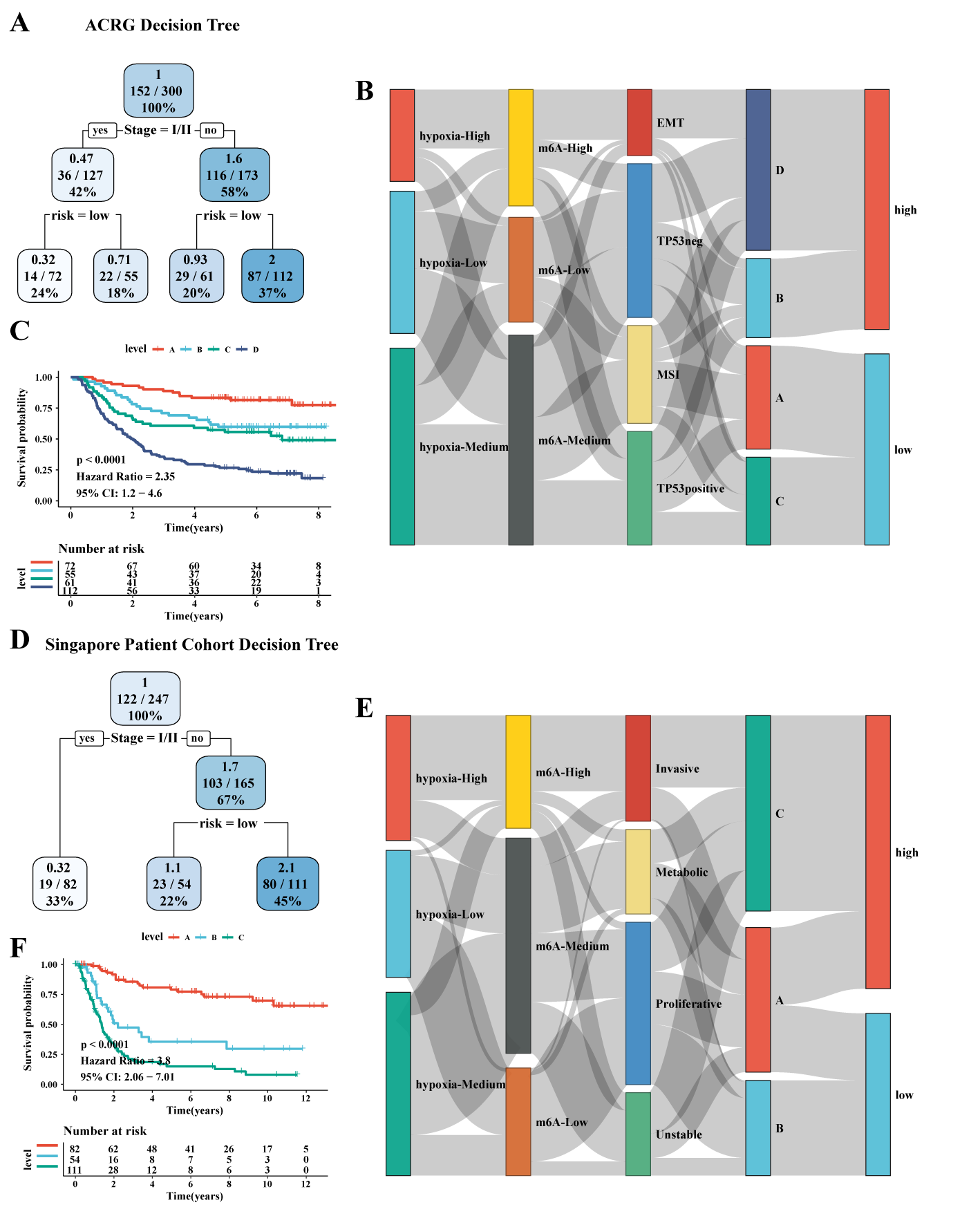

Supplement: Supplementary Figure 4 — Decision tree. (A) Simulating clinical decision of ACRG cohort subtypes. (B) Alluvial diagram was performed on hypoxiaCluster, m6Ascore, ACRG subtypes, decision cluster result, and risk clusters to analyze the mutual connection. (C) Kaplan–Meier curves for the decision cluster result from the ACRG decision tree. (D) Simulating clinical decision of Singapore patient cohort subtypes. (E) Alluvial diagram was performed on hypoxiaCluster, m6Ascore, Singapore patient, decision cluster result, and risk clusters to analyze the mutual connection. (F) Kaplan–Meier curves for decision cluster result from Singapore patient decision tree. [file Image_4.tif]
